# Monitoring health and nutrition claims on food labels in Brazil

**DOI:** 10.3389/fnut.2024.1308110

**Published:** 2024-02-07

**Authors:** Carolina Kikuta, Camila Aparecida Borges, Ana Clara Duran

**Affiliations:** ^1^Graduate Program in Public Health, Faculty of Medical Sciences, University of Campinas, São Paulo, Brazil; ^2^Center for Epidemiological Studies in Nutrition and Health, University of São Paulo, São Paulo, Brazil; ^3^Center for Food Studies and Research, University of Campinas, Campinas, Brazil

**Keywords:** health claims, nutrition claims, food packaging, food labeling, secondary data analysis

## Abstract

**Introduction:**

The monitoring of nutrition and health claims on food and beverage labels has been proposed by international and national organizations because it can collaborate with the development of public policies to regulate food labeling and marketing strategies. One way of carrying out this monitoring is by using data collected by private companies.

**Objective:**

To compare information on nutrition and health claims available in a commercial database of a private company that monitors the launch of new foods and beverages in Brazilian food retailers with information on those same claims manually coded by trained research assistants.

**Methods:**

This is a cross-sectional observational study using a data sample of newly launched food and beverages available at a commercial database from 2018 to 2021. We compared the information on health and nutrition claims available on the commercial dataset with reliable information on the same nutrition and health claims manually coded by trained research assistants using a tested taxonomy to classify such claims. We used Gwet’s Kappa AC1 with 95% CI and percentage agreement to compare both data sources and calculated sensitivity and specificity of the compared data.

**Results:**

A total of 6,722 foods and beverages were analyzed. Mintel-GNPD presented 36.28% (*n* = 2,439) of nutrition claims, while in the trained researchers’ coding, it was 33.73% (*n* = 2,267). We found a prevalence of 5.4% (*n* = 362) for health claims in Mintel-GNPD and 10.8% (*n* = 723) in the researchers’ coding. All subcategories of nutrition and health claims showed high agreement (Kappa >0.81). Health claims presented kappa = 0.89 with 33.7% sensitivity and 98.0% specificity while nutrition claims showed kappa = 0.86 with 92.9% sensitivity and 92.5% specificity.

**Conclusion:**

Nutrition and health claims showed high agreement, with great results in nutrition claims, indicating that Mintel-GPND is suitable for monitoring such claims on food and beverage packaging in Brazil. Additionally, our findings show a high prevalence of nutrition and health claims on food packages launched in the Brazilian food retail, highlighting the need to monitor these to develop public policies to regulate food marketing on packaging in Brazil.

## 1 Introduction

According to the World Health Organization, marketing to promote food and non-alcoholic beverages can be defined as any form of commercial communication or message that is designed to or has the effect of increasing the recognition, appeal, or consumption of specific food products (1). Marketing strategies can be made in different ways and using different marketing techniques, such as industry-sponsored advertisements, actions at points of sale, product design, and packaging (1). These strategies often include health and nutrition claims that highlight the nutritional content of the product, focusing on macronutrients and micronutrients and their potential health benefits in different age groups (2–4). The presence of nutrition and health claims on food packages can create a ‘health halo’ (5, 6), which is when an aspect of the product is highlighted as healthy and leads the consumer to understand the whole product as healthy, generalizing the one positive attribute and resulting in misconceptions and increasing the perceived healthiness of the food resulting in the consumption of ultra-processed foods that are harmful to health (7).

There are increasing sales of ultra-processed foods around the world, especially in low-and middle-income countries such as Latin America (8, 9). Food and beverages sold in retail outlets around the world, especially ultra-processed foods (10, 11), contain different types of marketing strategies to attract consumer attention and encourage purchase and consumption (2). Marketing strategies, such as health and nutrition claims, on unhealthy food packages high in added sugar, saturated fat, and sodium can influence and modify eating patterns leading to an unhealthy lifestyle, especially among children (12–15), which can be related to the development of non-communicable diseases (NCD) and obesity (16, 17).

Regarding the variety of strategies used by the food industry, according to INFORMAS (International Network for Food and Obesity/Non-communicable Diseases (NCDs) Research, Monitoring and Action Support), we can define nutrition and health claims as those that represent that there is a relationship between the food or ingredient and health and those that represent that food has a nutritional property due to its macro or micronutrient content, respectively (4). Some examples of health claims are ‘contains calcium which helps prevent osteoporosis’ or ‘helps lower cholesterol’. Nutrition claims can be represented by ‘source of vitamins and minerals’, ‘low in sugar’, and ‘high in protein’.

In Brazil, nutrition and health claims have been found on food packages of different types of food categories with different nutritional compositions. In a study carried out in Brazil (18), more than 80% of products including breakfast cereals and granola bars, fruit juices and nectars, and flavored drinks groups had claims on the package, with 28.5% having nutrition claims, followed by 22.1% having health claims. Another study also carried out in Brazil (11), found a prevalence of 33.3% of health claims on food packages and 32.1% of nutrition claims, 59.8% of which were ultra-processed products with at least one promotional strategy.

Public policies to regulate food marketing have been identified as one effective strategy that governments should invest in to promote healthier food environments and food choices that promote health, especially for children (19, 20). Evidence-based public policies are proving to be more effective in promoting long-term changes in the food environment, especially for the control and prevention of obesity and changes in eating patterns (21).

Monitoring strategies such as nutrition and health claims on food packages used by the food industry in a wide variety of environments (television, social media, internet, games, labels) has been proposed by international (22, 23) and national (24) organizations to support the development and improvement of public policies related to food labeling and marketing of foods considered unhealthy. Despite the great importance of monitoring these strategies, studies point to the difficulty of collecting data and generating up-to-date and reliable databases (25). In Brazil, there are no public databases on food and beverage packages to enable the monitoring of health and nutrition claims and their changes over time. One possibility to monitor and evaluate nutrition and health claims is through primary data collection that already has been made in Brazil (11, 18) and Chile (26), for example, collecting data through photos of food packages in supermarkets using a validated method (27). However, this process generally involves the use of multiple high-quality equipment, requires trained human resources to recognize package claims, and uses a large amount of financial resources and, it takes a lot of time to do the process (27). Considering that the food industry incorporates new products into the market in a very quick way, using different technologies and sources to do this kind of research is a possibility. One resource that has been used to monitor food labeling information is commercial databases developed by intelligence data companies and used by retailers and the food industry. The databases contain historical data on product launches of different countries, such as data from Mintel (28–30), Kantar, and others.

However, the databases of food packages from these types of companies contain hundreds of attributes that are not always organized for use in research and public policy. Especially in Latin America, where different countries are implementing labeling regulations to make healthier choices easier, it is important to have food labeling data to evaluate the regulation’s attributes and its impacts (31), such as product reformulation (32) and changes in sales of unhealthy foods (33). Therefore, this study aims to compare information on nutrition and health claims available in a commercial database of a private company that monitors the launch of new foods and beverages in Brazilian food retailers with information on those same claims manually coded by trained research assistants.

## 2 Materials and methods

### 2.1 Study design and sampling

This is a cross-sectional observational study using a data sample of newly launched food and beverages available at a commercial database from 2018 to 2021. We compared the information on health and nutrition claims available on the commercial dataset with reliable information on nutrition and health claims manually coded by trained research assistants using a tested taxonomy to classify such claims. The commercial database used is the Global New Products Database (Mintel-GNPD). Mintel is a private company that monitors the launch of new retail products in more than 80 countries around the world. The company also has information on other products such as cleaning and hygiene products among others (34).

In the Mintel-GNPD, the variable “*product description”* (e.g.: Creamy Chocolate Dessert has been repackaged, featuring an offer to pay less for more products. This gluten-free product has been inspected by the Brazilian Ministry of Agriculture, and retails in a 720 g pack with two 360 g units, each containing four 90 g tubs) and the variable “*claims”* (e.g.: “Economical, Gluten-Free, Allergens (Low/Reduced/No)”) hold much of the label’s possible nutrition and health claims. So, we selected these variables to compare. We downloaded the food images from the Mintel web platform to help us in identifying health and nutrition claims by trained researchers with a taxonomy method. Data collection, treatment, and coding of Mintel’s GNPD data will be detailed in the next sections.

We used data on foods and beverages available in the Mintel-GNPD database between 1 January 2018 to 31 December 2021. For sampling, we selected foods and beverages that represented the best-selling brands in Brazil according to the information provided by Euromonitor in the market share of foods and beverages sold in Brazil in the same period of the data available in Mintel (35). We included all foods and beverages from brands that together share up to 80% of sales in each of the available food categories in the Euromonitor data totaling 5,601 items. We also selected food brands owned by the largest Brazilian food retailers as Casino, Carrefour, WMB, Supermercados Cencosud, Supermercados BH, Cia Zaffari, and Supermercado Dia resulting in 1,456 products. Imported products or products with illegible images (*n* = 256), products from 2022 (*n* = 89), and infant formulas (*n* = 34) were excluded from the analysis. In the end, we had 6,722 food and beverage products evaluated. Using this sample, we analyzed Mintel’s GNPD variables of nutrition and health claims and package images.

### 2.2 Organization and coding of nutrition and health claims variables from Mintel-GNPD

The “*product description*” and “*claims*” variables are available in textual formats in the Mintel-GNPD database. The extraction of information about health and nutrition claims was done by developing a code in Stata that created dichotomous variables identifying the presence (1) or absence (0) of terms and expressions characterizing nutrition and health claims through quantitative content analysis (36). Quantitative content analysis is a research technique most used in communication research that examines symbols of communication and assigns them to numeric values according to valid measurements (35, 36).

To characterize and classify the nutrition and health claims in subcategories and subtypes, we used the protocol developed by the International Network for Food and Obesity/Non-communicable Diseases (NCDs) Research, Monitoring and Action Support (INFORMAS) (4), as shown in Table 1.

**Table 1:** Classification of health and nutrition claims according to INFORMAS “Food Labeling” protocol.

Claim categories	Subcategories	Subtypes
Health claims	General health claims	General, super, healthy Low GI/energy density Digestive health Bones health Oral health
	Reduction of disease risk	Heart-related claims Cardiology Society Nutrient absorption Cholesterol absorption Diabetes/Glycemic impact Osteoporosis Digestive health
	Nutrient and other function claim	Nutrient and muscle Nutrient and bone Nutrient and digestion Nutrient and immunity Nutrient and brain Nutrient and general health Nutrient and growth Nutrient and energy Nutrient and absorption Nutrient and strength
Nutrition claims	Health-related ingredient claim	
	Nutrient content claim	Fiber Energy Antioxidants/vitamins/minerals Fats Saturated fats Trans fats Omega 3 Omega 6 Sugar Protein Salt Cholesterol Taurine/guanine Caffeine
	Nutrient comparative claim	Reduced fat Reduced saturated fat Reduced trans fat More calcium Less salt Reduced sugar Reduced calories More fiber Reduced carbohydrates More protein Reduced cholesterol

A conference analysis was carried out to evaluate the developed code in Stata that identified claims in Mintel’s GNPD database. For this analysis, a sample of package images was evaluated individually to check whether the claim identified by the code was present on the label or not. For subtypes that resulted in up to 50 products identified in Mintel’s GNPD database, all images were evaluated, and for subtypes with more than 50 products identified, a sample of at least 20% of the products was evaluated.

### 2.3 Organization and coding of nutrition and health claim variables from food images

Mintel’s GNPD also contains images of all the sides of food packages. These images were used to code the nutrition and health claims by five nutritionists, who were trained to identify and code this information in a standardized way and the data were used for comparison with the data from Mintel-GNPD. This process occurred between December 2021 and July 2022. We developed a coding manual specially for this coding, also based on the INFORMAS protocol. The researchers did a one-month training before the coding and weekly meetings with the coordinator and supervisor of the research to clarify any doubts. We did an inter-and intra-rater analysis before starting the coding process to assess the reliability of the protocol questions and after finishing the coding process, both using 10% of the sample. Health claims showed Kappa = 0.96 on test–retest analysis and Kappa = 0.98 on inter-rater reliability and nutrition claims showed Kappa = 0.96 on test–retest and inter-rater analysis, which shows high agreement and high reliability of data coded by our researchers’ team.

### 2.4 Statistical analyzes

For the descriptive analyzes, absolute and relative distribution tables of the categories and subcategories of nutrition and health claims were created for both the data coded by the researchers and the data available in the variables proposed in Mintel’s GNPD database. We also described the food categories in which nutrition and health claims were most frequent for both sources of data.

To study the nutrition and health claims we compared the coding of Mintel’s GNPD variables with the coding done by our team of researchers using the images of all sides of the packages also available on Mintel-GNPD. For each category of claims and its subcategory and subtype, the agreement between both data sources was analyzed using Gwet’s Kappa AC1 and 95% Confidence Interval. Gwet’s measure was chosen because we are working with dichotomous variables and the measure it’s suitable for the analyzes. Additionally, it tends to be more stable dealing with substantial prevalence differences between the values of 0 and 1 (37, 38). We also calculated the percentage of agreement and 95% confidence interval. To interpret the Gwet’s Kappa AC1 values obtained, we used the scale proposed by Landis and Koch (39), where: less than 0.0, poor agreement; from 0 to 0.20, slight agreement; from 0.21 to 0.40, fair agreement; from 0.41 to 0.60, moderate agreement; from 0.61 to 0.80, substantial agreement; and from 0.81 to 1.00, high agreement. To help interpret the agreement between the datasets evaluated, Gwet’s Kappa AC1 and percent of agreement are presented with sensitivity and specificity.

To report sensitivity and specificity another analysis was carried out for summary statistics in a 2 × 2 table. We calculated sensitivity as the fraction of products identified with claims by the researchers and found on Mintel’s GNPD database and specificity as the fraction of products identified with no claims by the researchers and corrected identified with no claims on Mintel’s GNPD database. All statistical analyzes were carried out using Stata software version 15.

## 3 Results

A total of 6,722 foods and beverages available in Mintel’s GNPD database were analyzed from 2018 to 2021. Table 2 shows the comparison between the prevalence of nutrition claims by food groups present on Mintel’s GNPD database and coded by researchers using the food images, in these case we showed that the dairy products presented the higher prevalence of nutrition claims, with 24.03% (*n* = 586 vs. 23.03% *n* = 522 in researcher’s coding), followed by baked goods with 13.78% (*n* = 336 vs. 14.12% *n* = 320) and juices and fruit drinks with 10.25% (*n* = 250 vs. 10.94% *n* = 248) j. Table 2 also shows the prevalence of health claims by food groups. In Mintel’s GNPD database were found in 23.71% (*n* = 87) of the dairy products group (vs. 22.68% *n* = 164 in Researcher’s coding), followed by 14.44% (*n* = 53 vs. 11.62% *n* = 84 in Researcher’s coding) in baked goods and breakfast cereals (13.35% *n* = 49 vs. 8.44% *n* = 61 in Researcher’s coding). In the researchers’ coding, we also found a high prevalence of health claims of 10.24% (*n* = 74) in chocolates.

**Table 2:** Characterization (*n* and %) of the nutrition and health claims available on Mintel-GNPD and data coded by trained researchers according to different food groups.

Food groups	Nutrition claims				Health claims			
	Mintel-GNPD		Researchers’ coding		Mintel GNPD database		Researchers’ coding	
	*n*	%	*n*	%	*n*	%	*n*	%
Side dishes	54	2.21	39	1.72	9	2.45	15	2.07
Sweeteners and sugar	1	0.04	0	0.00	0	0.00	1	0.14
Baby food	55	2.26	45	1.99	16	4.36	38	5.26
Snacks	128	5.25	123	5.43	13	3.54	37	5.12
Sweets and chewing gum	55	2.26	53	2.34	20	5.45	25	3.46
Isotonic and energy drinks	32	1.31	28	1.24	30	8.17	9	1.24
Meal substitutes beverages	74	3.03	67	2.96	3	0.82	11	1.52
Ready-to-drink beverages	33	1.35	32	1.41	2	0.54	5	0.69
Hot drinks	13	0.53	11	0.49	7	1.91	19	2.63
Breakfast cereals	101	4.14	96	4.23	49	13.35	61	8.44
Fruits and vegetables	49	2.01	49	2.16	11	3.00	18	2.49
Processed fish. Meat and egg products	130	5.33	105	4.63	10	2.72	26	3.60
Dairy products	586	24.03	522	23.03	87	23.71	164	22.68
Sauces and condiments	206	8.45	187	8.25	4	1.09	33	4.56
Chocolates	133	5.45	155	6.84	8	2.18	74	10.24
Baked goods	336	13.78	320	14.12	53	14.44	84	11.62
Sweet breads fillings	42	1.72	48	2.12	23	6.27	11	1.52
Savory bread fillings	2	0.08	2	0.09	0	0.00	0	0.00
Meals	28	1.15	29	1.28	8	2.18	12	1.66
Soft drinks	56	2.30	47	2.07	0	0.00	0	0.00
Desserts and ice cream	56	2.30	47	2.07	5	1.36	25	3.46
Soups	7	0.29	8	0.35	0	0.00	12	1.66
Juices and fruit drinks	250	10.25	248	10.94	9	2.45	43	5.95
Water	12	0.49	6	0.26	0	0.00	0	0.00
*Total*	2,439	100	2,267	100	367	100.00	723	100.00

Table 3 shows the prevalence of claims found by category, subcategories, and subtypes of health claims. Health claims were classified in 5.46% (*n* = 367 vs. 10.76% *n* = 723 in Researchers’ coding) of the products in the Mintel-GNPD database. There was a higher prevalence of general health claims, with 72.75% (*n* = 267 vs. 90.73% *n* = 656 in Researchers’ coding), with a higher prevalence of the ‘general, super, healthy’ subtype (67.13% *n* = 243 vs. 86.03% *n* = 622 in Researchers’ coding). The reduction of disease risk claims (18.51% *n* = 67 vs. 4.56% *n* = 33 in Researchers’ coding) presented a higher prevalence of diabetes claims (12.98% *n* = 47 vs. 2.07% *n* = 15 in Researchers’ coding) and the nutrient and other function claims (33.15% *n* = 120 vs. 11.76% *n* = 85 in Researchers’ coding) showed a higher prevalence of nutrient and digestive health claims (12.43% *n* = 45 vs. 1.24% *n* = 9 in Researchers’ coding).

**Table 3:** Sensitivity, specificity, percentage of agreement and kappa for verifying the agreement of health claims in the Mintel-GNPD.

Claims	Mintel’s GNPD database (%)	Researcher’s coding (%)	Sensitivity (CI 95%)*	Specificity (CI 95%)*	Percent of agreement*	Gwet’s AC1 (CI 95%)*
*Health claims*	5.39	10.76	34.44% (32.62–34.88)	98.03% (97.70–98.36)	91.12	0.89 (0.88–0.90)
General health claims	77.62	90.73	26.83% (25.77–27.89)	98.27% (97.96–98.58)	91.3	0.90 (0.89–0.90)
General, super, healthy	67.13	86.03	23.95% (22.93–24.98)	98.38% (98.07–98.68)	91.49	0.90 (0.89–0.91)
Digestive health	3.31	1.80	76.92% (75.92–77.93)	99.97% (99.93–100.00)	99.93	0.99 (0.99–0.99)
Bones health	1.10	0.41	66.67% (65.54–67.79)	99.97% (99.93–100.00)	99.96	0.99 (0.99–1.00)
Oral health	1.66	2.21	31.25% (30.14–32.36)	99.99% (99.96–100.00)	99.82	0.99 (0.99–0.99)
Low GI/energy density	0.55	1.66	16.67% (15.78–17.56)	100.00% (100.00–100.00)	99.85	0.99 (0.99–0.99)
Reduction of disease risk	18.51	4.56	90.91% (90.22–91.60)	99.45% (99.27–99.62)	99.4	0.99 (0.99–0.99)
Heart-related claims	2.21	0.97	57.14% (55.96–58.33)	99.94% (99.88–100.00)	99.9	0.99 (0.99–0.99)
Cardiology Society	0.00	0.00	– (−)	– (−)	–	– (−)
Nutrient absorption	0.28	0.00	– (−)	– (−)	99.99	0.99 (0.99–1.00)
Cholesterol absorption	3.59	1.80	92.31% (91.67–92.94)	99.99% (99.96–100.00)	99.97	0.99 (0.99–1.00)
Digestive health	0.00	0.14	– (−)	– (−)	99.99	0.99 (0.99–1.00)
Osteoporosis	0.55	0.28	100.00% (100.00–100.00)	100.00% (100.00–100.00)	100.00	1.00 (1.00–1.00)
Diabetes/Glycemic impact	12.98	2.07	93.33% (92.74–93.93)	99.51% (99.34–99.68)	99.49	0.99 (0.99–0.99)
Nutrient and other function claim	33.15	11.76	72.94% (71.88–74.00)	99.13% (98.90–99.35)	99.8	0.98 (0.98–0.99)
Nutrient and energy	5.25	2.07	60.00% (58.83–61.17)	99.85% (99.76–99.94)	99.76	0.99 (0.99–0.99)
Nutrient and strength	4.70	1.38	80.00% (79.04–80.96)	99.87% (99.78–99.95)	99.84	0.99 (0.99–0.99)
Nutrient and general health	4.97	2.07	46.67% (45.47–47.86)	99.84% (99.74–99.93)	99.72	0.99 (0.99–0.99)
Nutrient and muscle	6.63	2.35	88.24% (87.47–89.01)	99.87% (99.78–99.95)	99.84	0.99 (0.99–0.99)
Nutrient and bone	5.8	1.94	92.86% (92.24–93.47)	99.88% (99.80–99.96)	99.87	0.99 (0.99–0.99)
Nutrient and growth	4.14	1.38	70.00% (68.90–71.10)	99.88% (99.80–99.96)	99.84	0.99 (0.99–0.99)
Nutrient and brain	1.10	0.28	100.00% (100.00–100.00)	99.97% (99.93–100.00)	99.97	0.99 (0.99–1.00)
Nutrient and digestion	12.43	1.24	100.00% (100.00–100.00)	99.46% (99.29–99.64)	99.46	0.99 (0.99–0.99)
Nutrient and immunity	2.76	1.66	83.33% (82.44–84.22)	100.00% (100.00–100.00)	99.97	0.99 (0.99–1.00)
Nutrient and absorption	0.83	0.14	0.00% (0.00–0.00)	99.96% (99.90–100.00)	99.94	0.99 (0.99–1.00)

*Data compared between Mintel’s GNPD database and trained researchers’ coding.

Table 3 also shows the results obtained for the analyzes of the agreement of health claims. The three subcategories – general health claims, reduction of risk disease, and nutrient and other functions – showed a Gwet’s Kappa ≥0.90 as well as all their subtypes. The sensitivity for the category was 33.75% and the specificity was 98.03%. Claims of reduction of disease risk about osteoporosis showed perfect agreement (Gwet’s Kappa = 1.00), with 100% sensitivity. The lowest sensitivity was observed for general health claims about low glycemic index (16.67%) presenting 100% specificity. Claims about the Cardiology Society, nutrient absorption, and digestive health from the subcategory of reduction of disease risk did not present sufficient data for sensitivity and specificity analysis but these last two subtypes showed high agreement.

Table 4 shows the prevalence of claims found by category, subcategories, and subtypes of nutrition claims in the Mintel-GNPD database and researchers’ coding, in Mintel’s GNPD database we found 36.28% (*n* = 2,439 vs. 33.73% *n* = 2,267 in Researcher’s coding) of nutrition claims. Nutrient content claims were the most frequent claims in the Mintel-GNPD data, with a prevalence of 78.88% (*n* = 1924 vs. 76.56% *n* = 1735 in Researcher’s coding) and there was a higher prevalence of vitamin and minerals claims (31.61% vs. 30.26% *n* = 686 in Researcher’s coding) in this subcategory. In the nutrient comparative claim (12.30% *n* = 300 vs. 17.16% *n* = 389 in Researcher’s coding), the highest prevalence was for reduced sugar claims (3.81% *n* = 93 vs. 3.79% *n* = 89 in Researcher’s coding) and reduced sodium claims (3.61% *n* = 88 vs. 4.81% *n* = 109 in Researcher’s coding). For the last subcategory, health-related ingredient claims (28.82% *n* = 703 vs. 35.42 *n* = 803 in Researcher’s coding), we found a higher prevalence for whole grain claims (12.96% *n* = 316 vs. 12.44% *n* = 282 in Researcher’s coding).

**Table 4:** Sensitivity, specificity, percentage of agreement and kappa for verifying the agreement of nutrition claims in the Mintel-GNPD.

Claims	Mintel’s GNPD database (%)	Researcher’s coding (%)	Sensitivity (CI 95%)*	Specificity (CI 95%)*	Percent of agreement*	Gwet’s AC1 (CI 95%)*
*Nutrition claims*	36.28	33.73	92.9% (92.28–93.51)	92.53% (91.90–93.15)	92.65	0.86 (0.85–0.87)
Nutrient content claim	78.88	76.53	95.5% (95.01–96.00)	94.65% (94.11–95.18)	94.87	0.91 (0.90–0.92)
Fiber	13.37	13.28	97.01% (96.60–97.42)	99.47% (99.30–99.64)	99.36	0.99 (0.99–0.99)
Energy	13.78	11.69	76.60% (75.59–77.62)	97.94% (97.60–98.28)	97.1	0.96 (0.96–0.97)
Antioxidants/vitamins/minerals	31.61	30.26	97.67% (97.31–98.03)	98.33% (98.02–98.63)	98.26	0.97 (0.97–0.98)
Fats	9.35	6.97	82.91% (82.01–83.81)	98.52% (98.23–98.81)	98.16	0.98 (0.97–0.98)
Saturated fats	1.72	1.46	100.00% (100.00–100.00)	99.87% (99.78–99.95)	99.87	0.99 (0.99–0.99)
Trans fats	6.15	6.40	95.86% (95.39–96.34)	99.83% (99.74–99.93)	99.75	0.99 (0.99–0.99)
Omega 3	2.09	2.16	95.92% (95.45–96.39)	99.94% (99.88–100.00)	99.91	0.99 (0.99–0.99)
Omega 6	0.86	1.01	86.96% (86.15–87.76)	99.99% (99.96–100.01)	99.94	0.99 (0.99–1.00)
Sugar	24.03	23.78	95.73% (95.25–96.22)	98.87% (98.61–99.12)	98.62	0.98 (0.98–0.98)
Protein	11.03	10.94	95.16% (94.65–95.67)	99.49% (99.32–99.66)	99.33	0.99 (0.99–0.99)
Salt	7.71	5.73	90.77% (90.08–91.46)	98.94% (98.69–99.18)	98.78	0.98 (0.98–0.99)
Cholesterol	4.63	4.19	100.00% (100.00–100.00)	99.73% (99.60–99.85)	99.73	0.99 (0.99–0.99)
Taurine/guanine	1.15	1.10	100.00% (100.00–100.00)	99.96% (99.90–100.00)	99.96	0.99 (0.99–1.00)
Caffeine	0.74	0.53	83.33% (82.44–84.22)	99.88% (99.80–99.96)	99.85	0.99 (0.99–0.99)
Nutrient comparative claim	12.30	17.16	62.98% (61.83–64.14)	99.13% (98.91–99.35)	97.04	0.96 (0.96–0.97)
Reduced fat	3.40	3.40	89.61% (88.88–90.34)	99.79% (99.68–99.90)	99.67	0.99 (0.99–0.99)
Reduced saturated fat	0.04	0.22	20.00% (19.04–20.96)	100.00% (100.00–100.00)	99.94	0.99 (0.99–1.00)
Reduced trans fat	0.08	0.09	0 (0.00–0.00)	99.97% (99.93–100.00)	99.94	0.99 (0.99–1.00)
More calcium	0.21	0.04	0 (0.00–0.00)	99.93% (99.86–99.99)	99.94	0.99 (0.99–1.00)
Less salt	3.61	4.81	75.23% (74.20–76.26)	99.91% (99.84–99.98)	99.91	0.99 (0.99–0.99)
Reduced sugar	3.81	3.79	82.56% (81.65–83.47)	99.67% (99.53–99.81)	99.51	0.99 (0.99–0.99)
Reduced calories	3.12	3.26	89.19% (88.45–89.93)	99.85% (99.76–99.94)	99.45	0.99 (0.99–0.99)
More fiber	0.25	0.13	33.33% (32.21–34.46)	99.93% (99.86–99.99)	99.73	0.99 (0.99–0.99)
Reduced carbohydrates	0.00	0.00	– (−)	– (−)	–	– (−)
More protein	0.16	0.04	100.00% (100.00–100.00)	99.96% (99.90–100.00)	99.96	0.99 (0.99–1.00)
Reduced cholesterol	0.00	0.09	– (−)	– (−)	99.97	0.99 (0.99–1.00)
Health-related ingredient claim	28.82	35.42	70.61% (69.52–71.70)	97.63% (97.27–98.00)	94.41	0.93 (0.92–0.93)
Wholegrain	12.96	12.44	97.16% (96.77–97.56)	99.35% (99.16–99.54)	99.26	0.99 (0.98–0.99)
Bacteria/culture/probiotics/prebiotics	1.15	4.72	96.26% (95.81–96.72)	99.37% (99.18–99.55)	99.32	0.99 (0.99–0.99)
Fruits	5.95	5.51	31.20% (30.09–32.31)	99.68% (99.55–99.82)	98.41	0.98 (0.98–0.98)
Nuts	0.08	0.44	20.00% (19.04–20.96)	100.00% (100.00–100.00)	99.88	0.99 (0.99–0.99)
Honey	0.16	0.66	13.33% (12.52–14.15)	99.97% (99.93–100.01)	99.78	0.99 (0.99–0.99)
Grains/seeds	2.09	4.32	28.57% (27.49–29.65)	99.65% (99.51–99.79)	98.62	0.98 (0.98–0.98)
Vegetables/plants	4.22	3.62	56.10% (54.91–57.28)	99.14% (98.92–99.36)	98.62	0.98 (0.98–0.98)
Milk/cream	5.82	3.00	39.71% (38.54–40.88)	98.27% (97.96–98.58)	97.68	0.97 (0.97–0.97)
Oils	3.65	1.54	60.00% (58.83–61.17)	99.90% (99.82–99.97)	99.69	0.99 (0.99–0.99)
Cocoa	2.46	4.46	71.29% (70.21–72.37)	99.74% (99.62–99.86)	99.32	0.99 (0.99–0.99)

*Data compared between Mintel’s GNPD database and trained researchers’ coding.

Table 4 also shows the agreement results of nutrition claims, which also showed high agreement with Gwet’s Kappa above 0.81 in the category. The category showed a Gwet’s Kappa of 0.86, classified as high agreement and all the subcategories also showed high agreement (≥ 0.90), as well as all the subtypes. The category had a sensitivity of 92.90% and a specificity of 92.53%. The nutrient content claims for saturated fats showed 100% sensitivity, as well as the nutrient claims for cholesterol and taurine and guanine. The nutrient comparative claims of higher protein also showed 100% sensitivity, on the other hand, comparative claims about reduced trans-fat and higher calcium showed 0% sensitivity although both resulted in Gwet’s Kappa =0,99 (high agreement). Among all categories, health-related ingredient claims about honey showed the lowest sensitivity (13.33%) but with 99.97% specificity. Comparative claims of reduction of carbohydrates did not present sufficient data for analyzes.

## 4 Discussion

This study provided a deep characterization of the nutrition and health claims present on packaged foods and beverages launched in Brazilian food retail between 2018 and 2021 and found in the Mintel-GNPD. We showed that this dataset could be a reliable alternative when the purpose is to monitor health and nutrition claims on food labels in Brazil. Nutrition claims were the most prevalent, with 35–40% of the claims in both data sources. We found that some food groups, as dairy products, baked goods, breakfast cereals, and isotonic and energy drinks had a higher prevalence of nutrition claims, and other food groups as dairy products, baked goods, juices and fruit drinks, and sauces and condiments had a higher prevalence of health claims, and in general the prevalence is higher in the Mintel-GNPD than data codified by trained researchers. We found high agreement in the information available in Mintel-GNPD for all categories and subcategories of nutrition and health claims evaluated, showing a high potential for using Mintel-GNPD to monitor these strategies on food packages. The high prevalence of nutrition claims on food packaging is concerning because it can influence and modify eating patterns, especially in children, and contribute to the development of non-communicable diseases (13, 16). Since Brazil does not yet have representative and up-to-date public data on the information on food and drink packaging sold in supermarkets, this study provides an alternative source of reliable data for monitoring and characterizing health and nutrition claims in the country.

The data evaluated in Mintel’s GNPD database showed a prevalence of 36.28% of nutrition claims on food packaging. Regarding the high prevalence of nutrition claims found in this study, other previous studies that have analyzed food and drink labels with data collected from supermarket shelves have also shown something similar, finding 28.5% of nutrition claims on food packaging (18). Another study that evaluated claims and marketing strategies in Brazil found 32.8% of nutrition claims and health claims and revealed a high prevalence of these claims on dairy products (40). Breakfast cereals, bakery products, and dairy products were also food groups found with the highest prevalences of promotional strategies (11), including nutrition and health claims, which corroborates our results. This shows that, in a way, the data available from Mintel-GNPD represents what Brazilians have been finding on supermarket shelves.

We found two times more health claims in the information collected and coded by trained researchers using food images and INFORMAS taxonomy than the variables extracted from Mintel-GNPD, and this difference had an impact on agreement and sensitivity measures. One possible reason is that the INFORMAS protocol used to classify health claims considers brand names, phrases below the brand name, and slogans as health claims, and in the Mintel-GNPD we did not use the variable “brand” to identify the claims (e.g., a product with the name Naturale was considered as a health claim). However, nutrition and health claims that most impact consumers and influence their food choices are highlighted on the front of the package (18, 41) and on other sides of the package as well (11, 42) and are not necessarily present in brand logos and slogans.

Studies evaluating the validity and reliability of the information available on secondary databases have been made through the years in epidemiology (43), as a way of certifying that variables, measures, or information collected by third parties can be used to generate good results in quantitative studies that associate health outcomes with risk factors, for example. Some examples of these studies are related to the food environment, comparing it to primary data collected, as made in the United States (44) and United Kingdom (45). A systematic review (46) evaluated the use of commercial databases in public health studies, including companies such as Euromonitor and Kantar, and concluded that these data can be useful tools with great potential for public health nutrition studies if used according to their limitations.

The inadequate use of nutrition and health claims on food packaging is concerning because these claims are used to persuade consumers to buy products, especially children (47). When there is no effective regulation the use of marketing strategies targeted at children can increase and mislead the consumers’ understanding (48). In addition, the lack of regulation makes it difficult to check the veracity of the claims, as has happened in Brazil where a brand of biscuits claimed to have honey on the package but none of them had honey on the ingredient list (49). The high frequency of nutrition and health claims on packages is concerning and highlights the need to monitor these strategies in a faster way, considering that the food industry incorporates new products into the market very quickly. These results emphasize the need to improve regulations and inspections on the use of nutrition and health claims to reduce the health halo effect on consumers and not be present in foods that are high in sugar, sodium, and fats. Besides these types of claims being regulated in Brazil (50–52), they are used as a form to promote the product, increasing the perception of healthiness by consumers, but are often present on foods that have poor nutritional quality (11, 18).

Therefore, by monitoring these strategies using a commercial database we can characterize the types of products that contain this health and nutrition claims, which types of claims are most prevalent, the target audience, the brands involved, and other factors that are key to developing and evolving regulatory aspects in Brazil. However, this characterization of health and nutrition through the Mintel database first needs an evaluation of its data, as a first step, since Brazil does not yet regulate marketing, we can explore the types of products and types of claims most used by ultra-processed food manufacturers.

This study has some limitations. Mintel’s GNPD database is updated with every launch or update – reformulation, packaging design, or any other modification – and for that reason, it is not possible to know if any product present in the database and analyzed in this research has been discontinued from the market. Nevertheless, the sample is large and includes approximately 20% of the products available for the period from 2018 to 2021. We included products from the best-selling brands in Brazil, which together hold an 80% market share of sales, as well as all private-label foods from seven Brazilian retail markets. To categorize the claims, we classified them into categories and subcategories. When classifying into subcategories, we found a very low prevalence of some claims, for example, the ‘nuts’ subcategory of health-related ingredients (below 0.5% in both datasets) that could have affected the sensitivity measure. Despite the limitations, this is the first study to compare information of nutrition and health claims present in Mintel-GNPD with manually coded claims by trained researchers. Another strength of the study is the use of the INFORMAS protocol to evaluate the nutrition and health claims. As it is a standardized international protocol, it makes it easier to compare results with other countries (4). Also, Mintel-GNPD has data from more than 80 countries and the coding that we developed in Stata can be applied to analyze nutrition and health claims from other countries too.

## 5 Conclusion

The results found in this study indicate that the information on health and nutrition claims available at Mintel-GPND is suitable for monitoring such claims on food and beverage packaging in Brazil. Monitoring the use of nutrition and health claims on food packages is central to developing and improving public policies to regulate food labeling, especially when it comes to children and adolescents. The considerable expenses and frequently extended timelines associated with gathering primary data, compounded by the absence of publicly accessible databases containing this information, present challenges in effectively surveilling these claims. Thus, verifying whether Mintel-GNDP is suitable for monitoring health and nutrition claims in Brazil will allow us to take advantage of available datasets to facilitate policy monitoring.

The development and improvement of public nutrition and health policies depend on the production of scientific evidence through monitoring the marketing strategies, such as health and nutrition claims, used by the food industry so they can act effectively to improve the diet of the Brazilian population, reducing the consumption of low nutritional quality foods and preventing the development of related diseases such as obesity and diabetes. Public policies aimed at regulating marketing strategies are a fundamental part of protecting the population’s health, as they aim to protect consumers from abusive marketing practices, provide clearer and better-quality information about the food they are buying, and offer tools for healthier food choices.

Having established that the information on health and nutrition claims available at Mintel-GNPD for Brazil allows for future research endeavors. These data can provide valuable information for monitoring and enforcement of the labeling legislation in Brazil.

## Data availability statement

The raw data supporting the conclusions of this article will be made available by the authors, without undue reservation.

## Author contributions

CK: Conceptualization, Formal analysis, Writing – original draft. CAB: Methodology, Writing – review & editing, Supervision. ACD: Methodology, Writing – review & editing, Supervision.
